# Determination of the inhibitory effects of N-methylpyrrole derivatives on glutathione reductase enzyme

**DOI:** 10.1080/14756366.2018.1520228

**Published:** 2018-10-26

**Authors:** Esma Kocaoğlu, Oktay Talaz, Hüseyin Çavdar, Murat Şentürk, Claudiu T. Supuran, Deniz Ekinci

**Affiliations:** a Kamil Ozdag Science Faculty, Karamanoglu Mehmet Bey University, Karaman, Turkey;; b Education Faculty, Dumlupınar University, Kütahya, Turkey;; c Pharmacy Faculty, Agri Ibrahim Cecen University, Agri, Turkey;; d Neurofarba Department, University of Florence, Firenze, Italy;; e Ondokuz Mayis University, Faculty of Agriculture, Department of Agricultural Biotechnology, Samsun, Turkey

**Keywords:** N-methylpyrrole, coupling, glutathione reductase, antimalaria, antioxidant

## Abstract

Glutathione reductase (GR) is a crucial antioxidant enzyme which is responsible for the maintenance of antioxidant GSH molecule. Antimalarial effects of some chemical molecules are attributed to their inhibition of GR, thus inhibitors of this enzyme are expected to be promising candidates for the treatment of malaria. In this work, GR inhibitory properties of N-Methylpyrrole derivatives are reported. It was found that all compounds have better inhibitory activity than the strong GR inhibitor N,N-bis(2-chloroethyl)-N-nitrosourea, especially three molecules, 8 m, 8 n, and 8 q, were determined to be the most powerful among them. Findings of our study indicates that these Schiff base derivatives are strong GR inhibitors which can be used as leads for designation of novel antimalarial candidates.

## Introduction

In aerobic organisms, free radicals are produced via normal reactions in the metabolism and can also be generated in the form of reactive oxygen species (ROS), such as superoxide anion radicals (O_2_^•−^), hydroxyl radical (^•^OH), hydrogen peroxide (H_2_O_2_), and etc. In the metabolism, equilibrium between the natural antioxidative defence system and ROS exists. If the equilibrium between ROS and antioxidant defence system stops working properly, the reactive oxygen species cause cell damage which then results in several diseases including cancer, cardiovascular diseases, age related degenerative diseases, arthritis, and diabetes^1–3^.

Glutathione reductase (GR) plays a critical role in gene regulation, maintenance of high rates of GSH/GSSG, intracellular signal transduction, clearing of free radicals and reactive oxygen species, and preservation of redox status of intracellular species and is an important enzyme in the cell. Under normal conditions, glutathione is mostly present in reduced form (GSH), yet it might be rapidly oxidized to GSSG as a response to oxidative stress response in order to protect the cell and cell components. However, glutathione reductase reduces GSSG to GSH with NADPH and the intracellular ratio of GSH/GSSG remains above 99%.

GSSG+NADPH+H+→2 GSH+NADP+

Because of the key function of GSH in numerous cellular processes, GSH level and GSH/GSSG ratio are associated with many human diseases such as cancer, cardiovascular diseases, diabetes, AIDS and Alzheimer. GSH is also used for the detoxification of haem and an increase in the amount of intracellular GSH is responsible for the development of the chloroquine resistance. In addition, glutathione reductase inhibitors have been found to possess antimalarial and anticancer activity^4–7^.

The reason for investigating Schiff’s base derivatives as GR inhibitors is the fact that simple molecules have been shown to be inhibitors of GR. Grellier et al. have reported the antiplasmodial activity of a number of homologous nitroaromatic compounds with either strong or weak inhibitors of GR. To this end, a new irreversible GR inhibitor 2-acetylamino-3-[4–(2-acetylamino-2-carboxyethyl sulfanyl thiocarbonyl amino) phenyl thiocarbamoyl sulfanyl] propionic acid (2-AAPA) was selected in this study and this study showed that 2-AAPA increased anticancer activity, NADPH/NADP+ and NADH/NAD+ ratios, increased GSSG and decreased GSH and inhibited yeast GR^8–11^.

**Figure 1.**
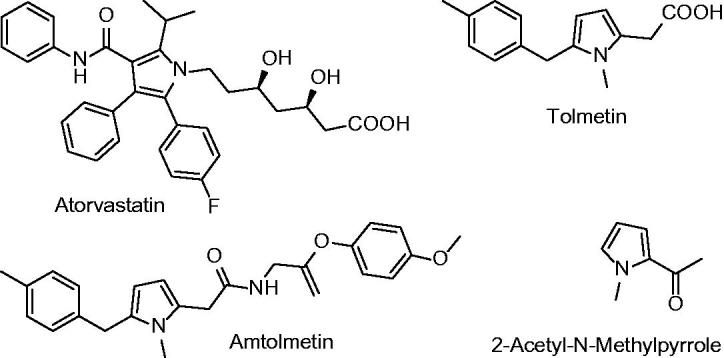
Pyrrole containing drugs.

The pyrrole ring, which is found in many natural products and used in many pharmacologically related and other functional syntheses, is one of the most important heterocyclic compounds (Figure 1). The pyrrole ring is available in a variety of drugs containing antituberculosis agents, analgesics, COX-2 inhibitors, immune system suppressants and antiinflammatory. In addition, 2-acetyl 1-methylpyrrole is the flavouring agent. 1,2,5 tri-substitution pattern pyrrole, displays distinct biological properties as shown by antiinflammatory agents antolmet and tolmetin. As mentioned above, this heterocyclic system is attractive scaffolding that confirms the use of chemical diversity for the purposes of medicinal chemistry^12–18^.

**Figure 2.**
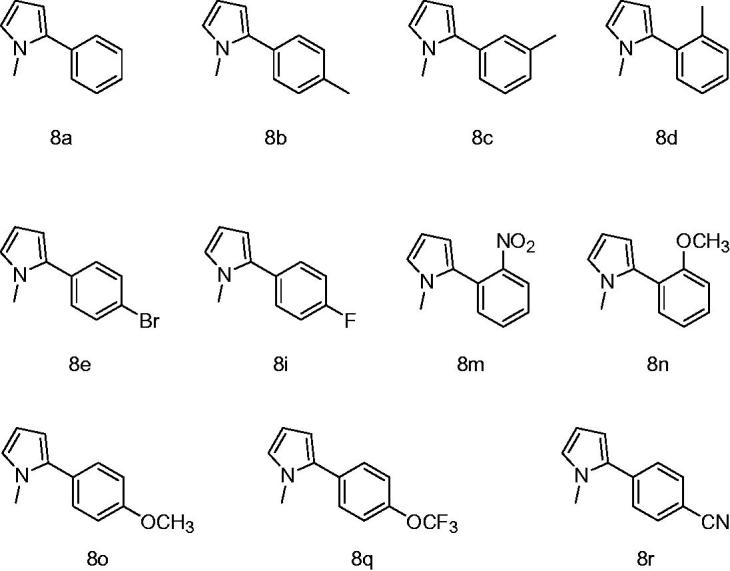
Chemical structures of tested compounds.

In this study, for the aim of designation of novel GR inhibitors, we have synthesized N-methylpyrrole derivatives and evaluated their ability to inhibit GR (Figure 2). The inhibition is reported as the IC50 values and the results are averages of at least three independent analyses.

**Figure 3.**
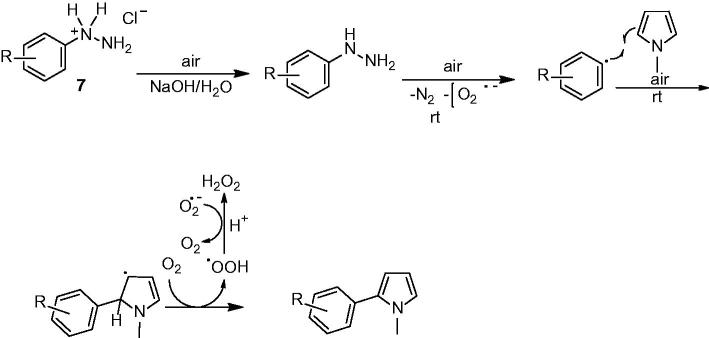
Synthesis pathway of the tested compounds.

## Experimentation

### Chemistry

#### General

All reactions were carried out in air. Anhydrous solvents were distilled prior to use with appropriate drying agents. Thin layer chromatography was performed on Merck silica gel 60 F_254_. Visualization was performed by means of UV light (254 nm) and by staining with ethanolic phosphomolybdic acid solution. NMR spectra were recorded using a Varian 200 MHz NMR instrument.

### General procedure for arylation of N-methyl pyrrole with phenylhydrazine hydrochloride salts

Six hundred and seventy milligrams pyrrole and 72 mg phenylhydrazine hydrochloride salt were reacted. Then 0.5 M NaOH was added dropwise over a period of 30 min. The resulting mixture was stirred at the room temperature for 50–60 h. Excess of pyrrole and water was evaporated with at room temperature, and the remaining solid was purified by flash column chromatography (EtOAc/hexane %25).

### Glutathione reductase inhibition

Activity of the glutathione enzyme was measured by Beutler’s method^22^ in which one enzyme unit is defined as the oxidation of 1 mmol NADPH per min under the assay condition at 25 °C, pH 8.0. Different concentrations of the inhibitors were applied to the enzyme solutions and all compounds were tested in triplicate at each concentration used^18–21^. Control cuvette activity was assumed as 100% in the absence of inhibitor. A graphic of activity-versus inhibitor concentration was drawn for each compound (Figure 3).

## Results and discussion

### Chemistry

Phenylhydrazine salts have been broadly used for modification of organic molecules with aryl groups. This synthetic procedure achieved transition metal free arylation of pyrrole in a eco-friendly way. The synthetic process started from the reaction of phenylhydrazine hydrochloride salt with NaOH to rapidly forma free phenylhydrazine, a slow oxidation with air to produce aryl radical^15^. Aryl radical X reacted with N-methyl pyrrole at room temperature to form allyl radical X which was supported by radical resonance after that losing a single electron loosing with air oxidation and eliminating a proton resulted acrylate pyrrole^16^.

### Arylation mechanism of N-methylpyrrole

**Figure 4.**
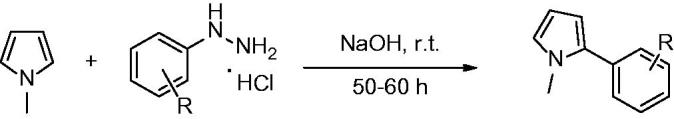
Reaction of arylhydrazine hydrochloride salts with N-methylpyrrole.

To obtain the biologically active target compounds N-methylpyrrole derivatives (**8a–r**), N-Methylpyrrole was reacted with different arylhydrazine hydrochloride salts in the presence of sodium hyroxyde as a catalyst. These reactions were in moderate yield (70–91%) under room temperature (Figure 4). The reaction times were from 50 to 60 hours^16^.

### Biological studies

In this work, we reported GR inhibitory capacity of N-methylpyrrole derivatives (8a-r). As known, glutathione reductase has been purified from various organisms and the influences of drugs, pesticides and various chemicals on GR activity have been investigated. In the current study, GR from baker's yeast (*S. cerevisiae*) was used and the inhibitory potentials of the synthetized compounds were determined using Beutler method with NADPH and GSSG as substrates and further kinetic studies were performed using the same method.

The data in Table 1 shows the relation between the compounds **8a–r** and glutathione reductase enzyme. The results are also compared with N,N-bis (2-chloroethyl) -N-nitrosourea which is a strong GR inhibitor and anticancer drug^23^.

**Table 1.** IC_50_ values obtained from regression analysis graphs for GR enzyme.

Compound	IC_50_ (µM)
**8a**	4.942
**8b**	1.402
**8c**	1.663
**8d**	1.050
**8e**	1.498
**8i**	1.422
**8m**	0.104
**8n**	0.678
**8o**	1.435
**8q**	0.846
**8r**	1.792
**N,N-bis(2-chloroethyl)-N-nitrosourea**	645

Mean from at least three determinations. Errors in the range of ±3% of the reported value (data not shown).

Our results showed that compound **8m** behaved as the strongest inhibitor against GR enzyme with the IC_50_ value of 0.104 µM. Second most powerful inhibition was observed by the structurally similar compound **8n** with an IC_50_ value of 0.678 µM. A very similar compound to **8n** is **8o** which showed much weaker inhibition (0.678 µM). This result is interesting because the only difference between these two molecules (8n and 8o) is the position of the metoxy group on the aromatic ring. Third most potent inhibitor was 8q with an IC_50_ value of 0.846 µM which includes electronegative flor atom. Remaining compounds showed similar inhibition values (1.402–1.792 µM) except for **8a** which exhibited the weakest inhibition with an IC_50_ value of 4.942 µM. Nevertheless, all of our compounds showed much more powerful inhibition than N, N-bis(2-chloroethyl)-N-nitrosourea which is a strong GR inhibitor in the literature^23^.

## Conclusions

Here we synthesized and evaluated the inhibition potential of a new class of GR inhibitors. Our compounds showed higher inhibition capacity than reference GR inhibitor and also than those of many drugs, metal ions, and other chemical compounds which have been tested for GR inhibition so far. Kinetic measurements allowed us to define N-methylpyrrole derivatives besides N,N-bis (2-chloroethyl) -N-nitrosourea as submicromolar-low micromolar inhibitors. This novel class of inhibitors might bind differently than any other known GR inhibitors and might be located between the glutathione binding sites in the enzyme cavity. As the inhibitors of GR are very important for both designation of antimalaria agents and other drugs, our findings provide useful data for further investigations in medicinal chemistry and pharmacology with the possibility to design novel molecules with higher inhibition potentials as compared to clinically used inhibitors.
